# Treatment of Diphtheria

**Published:** 1861-02

**Authors:** Wm. Henry Thayer


### TREATMENT OF DIPHTHERIA.

BY WM. HENRY THAYER, M.D., &c.

In the plan of treatment which finds general favor in France, England, and America, at the present time, several results are aimed at: to arrest the local affection, to sustain the patient, and eliminate the poison from the system. Nearly all physicians have concurred in the manner in which these several indications are to be answered. Some, however, consider that the disease may be successfully treated by local measures alone; that caustic applications made to the exudation and the inflamed mucous surface, at the outset, will be the best and all-sufficient treatment; believing that the affection of the throat is the nidus, from which the system at large is affected. Amongst the varieties of diphtheria, we may find some cases that are amenable to topical measures only, and this especially where its onset is gradual. Yet the disease sometimes sets in with sudden violence, with vomiting and purging, or convulsions, or the patient begins early to sink. In such cases, and probably in the very large majority of cases, exclusively local treatment cannot be depended upon.

Local and constitutional measures have been most generally combined, and with a reasonable degree of success.

The local measures consist of the use of caustics for the destruction of the false membrane, or antiseptics, which prevent its decomposition; and milder applications of caustic or astringent solutions for the surrounding parts, and the whole surface, after the removal of the exudation.

The local treatment of Bretonneau, in 1818, was a combination of strong hydrochloric acid, with three parts of honey, applied to the exudation. The same combination has been applied by many physicians since that time. The application should be made twice a day to the false membrane, until it is removed, and as often as it is renewed—to be omitted when the mucous membrane is exposed. After this, the usual plan is to apply an astringent, such as a solution of tannin or alum. The acid should be applied to the exudation with a camel's-hair pencil.

Another topical treatment, and perhaps the one in most general use, is that of nitrate of silver, which was first recommended for use in this disease by Dr. Mackenzie, of Glasgow, in 1825. It has the advantage of being much more manageable than hydrochloric acid. It is applied in the solid stick, or in strong solution. It is the practice of some to make an application of the stick to the exudation, and of a solution to the whole inflamed surface. It is very important, whatever strength be used, that every part of the reddened surface be reached by the solution. It is desirable to destroy, and remove, if possible, the false membrane, and cauterize the surface beneath; and to repeat the application daily, or twice a day, as long as a disposition to form an exudation continues. Dr. Wells, of Milwaukee, Wis., advises that the false membrane be removed with the forceps, and a solution of nitrate of silver subsequently applied. As the exudation begins to diminish, and the surface around it to improve, the strength of the solution should be diminished, and the applications should be less frequent. Some use the stick, more a solution, of the suitable strength of which there seems to be a great range of opinion—few using more than one drachm to the ounce of water, more not more than gr. xxx, and some as little as gr. xv. These weaker solutions may often be sufficient for the general reddened surface—but I doubt if they are of any value upon the exudation, or the surface beneath it. I prefer to apply the stick to the exudation, and a solution of 2 dr. to 1 oz. to the surrounding mucous membrane, for the early applications, to be omitted or diminished as the disease begins to yield.

*A great point in the treatment is to begin it early.* The success depends very much upon it. Evanson and Maunsell state that caustics are capable not only of "destroying the false membrane when formed, but of altering the morbid action going on, and so of arresting the progress of the disease," (before the exudation.) "It is a good and safe rule," say they, "in practice, to wash over the back of the throat with a strong solution of the nitrate of silver, the moment the mucous membrane appears affected, when a child sickens during the prevalence of scarlatina, diphtherite, etc. The case is thus not only made manageable, but the evil altogether averted."

Dr. Ramskill, of London, objects to the indiscriminate use of caustics. He thinks "the cases in which it does act well are those in which there is little effusion or swelling into the surrounding tissues, and under the diphtheritic patch, with no marked tendency to glandular engorgement." He believes that in some cases caustics have produced great enlargement of the glands, and aggravation of all the symptoms, and says, "in fact, in all cases where the first application of caustic has shown the tendency to excessive glandular enlargements, the local treatment cannot be too soothing and gentle." (*Lancet*, *Feb.* 1859.) It is a suggestion worthy of attention, but which has not, that I am aware of, been confirmed by the observation of others. The only other objection to the use of caustics, that I have met with, is from Mr. Bristowe, and on purely theoretical grounds.

The remaining applications most in favor are Tincture of the Sequichloride of Iron, Chlorate of Potash, Chloride of Soda, of Lime, and of Sodium, or various combinations of them, as in the Chlorine Mixture, which is composed of Hydrochloric Acid and Chlorate of Potash. Alum, Borax, and Tannin, have also their advocates.

The Tincture of the Sequichloride of Iron is applied in full strength with the brush. Chlorate of Potassa is used in saturated solution, which is about gr. xviii to the ounce; the Chloride of Soda, in "Labarraque's Solution," diluted to the strength of 1 dr. to two ounces; and the Chloride of Sodium has been used in strong solution, or with vinegar—a form which has long been a popular gargle, in inflammation of the throat. These various medicines are used as gargles—the most uncertain method, and out of the question with children—or are applied with a camel's-hair pencil, or by means of a syringe.

Mr. Hutchinson, of London, illustrates the value of Chlorate of Potash as a local application, in a case of buccal diphtheria—of which the following is an abstract. A pallid, delicate boy, eight years old, was admitted to the Metropolitan Free Hospital—with his right cheek greatly swollen, tense, and hot—lined with a diphtheritic exudation, and with gums inflamed, and bleeding at the slightest touch, and very fœtid odor to the breath. This condition had existed a fortnight. "It is well known that for the form of stomatitis above described, chlorate of potash, in full doses, is almost a specific. Wishing to ascertain whether its virtues are due to its local or its constitutional influence, Mr. Hutchinson ventured to direct that it should be used only topically." It was carefully and thoroughly used as a wash every two hours, and no other treatment employed. At the end of a fortnight the mouth was well. The boy still looked well, and steel wine was prescribed.

Mr. Hutchinson very pertinently asks, "Why not try the chlorate as a topical application in cases of pharyngeal and tracheal diphtheria?"

It cannot fail to be noticed, that many of the remedies employed in diphtheria, are combinations of chlorine. And all the various compounds appear to have been adopted without any preconceived notion of the special applicability of this element to the disease, but by experience with them in a variety of affections, some of which are kindred to it. Thus, the Tr. Ferri Sesquichlor. was adopted for its excellence as a topical application in erysipelas; the chloride of soda and of lime, as useful applications for foul ulcers; the chlorate of potassa, from its successful administration in aphthous stomatitis; and chloride of sodium with vinegar, from its use in tonsillitis. There is same analogy of Mr. Hutchinson's results, in the abortive treatment of small pox by repeated daily ablutions with chlorine water, as described in Champonnierre's Journal of Medicine and Surgery, Art. 5627.

The data for forming an opinion of the value of local treatment are often unreliable, from the imperfect manner of applying the wash to the fauces. This is especially the case when we depend on gargles, or leave applications to be made by a nurse. They should be directed in such forms as to insure their thorough use, if necessarily delegated to nurses. In a case of severe diphtheritic inflammation after scarlet fever in the spring of 1859, it extended through the fauces, the pharynx, and the posterior nares, with great swelling, and a considerable semipurulent discharge from the nostrils. I made thorough application of Labarraque's Solution, diluted, several times daily, by means of a syringe, throwing a stream through the nostrils, and also through the fauces. It is less painful and less troublesome to a child than the same application made with sponges, and is far more thorough. The nose and throat are cleaned of pus and mucus, and loose exudation membrane, which are coughed, vomited, or spit up, at the same time that the wash is a curative application to the inflamed surface.

Dr. Lawrence, of North Adams, says, "I relied on the inhalation of steam as a local application, except in two cases, when I used the nitrate of silver. Steam has many advantages over the caustic, as the child does not object to breathing it, and consequently is not disturbed. In one case where the throat so far as could be seen was blanketed with the peculiar membrane, and suffocation seemed imminent, I was gratified to see the whole thrown off entire by the sickness caused by the inhalation of steam. The breathing was at once relieved, and the child recovered." I have no doubt, from experience in the employment of a constant inhalation of steam in membranous croup, of its great value in cases of diphtheria; but, whether it can safely supersede the caustic in grave epidemics, remains to be proved. The cases in North Adams were not of the gravest form: in eighty-one cases there was no death, and not one had any form of paralysis as a sequel. Dr. Lawrence's experience is nevertheless very instructive and valuable.

After the adoption of a course of active local treatment, two other indications remain to be answered: to sustain the patient and eliminate the blood-poison.

In the attainment of these objects, there is entire unanimity in the medical profession. The disease is everywhere recognized as one to which antiphlogistic treatment is inapplicable; but a steady tonic and stimulating course with the most nutritious food, constitutes the usual course of general treatment, and that which will not only best sustain the patient, but restore the normal condition of the blood.

The compounds of chlorine administered internally seem to be as well employed as in topical applications. The chlorate of potassa alone, or in combination with hydrochloric acid (in the "chloric mixture,") is the most used. It should be given in full doses, frequently repeated—ten grains every four hours for an adult, and a proportional amount for children. But it need not be limited to this quantity, if a larger amount can be administered. Chlorate of potash has an advantage over the tincture of the sesquichloride of iron, as an alkali—in its resolvent effect on the fibrine of the blood, and hence a probability of its diminishing and arresting the exudation. If, as is possible, it answers this purpose as well as mercurials, it is undoubtedly better that it should be employed in preference to them.

I believe that there is a very general inclination at the present time to treat membranous croup (a sthenic disease) without mercurials. If, then, medical men have arrived at the conclusion that their success is greater in the treatment of that affection without calomel, there appears to be a still stronger objection to employing it in a disease of so low and malignant a type as diphtheria.

It would not be fair to omit the evidence of Conolly in favor of mercurials, who with Bretonneau and Guersent advocated their use, and reported that their employment had been followed by great success. "To insure its success," says Tweedie, on Conolly's authority, "the full mercurial influence is necessary." At the same time, it must be remembered that the testimony was given thirty-five years ago, since when the disease itself has become more adynamic; and that the general recommendations of mercury by these gentlemen must be received with the allowance which should always be made for the admiration of a new remedy; it was a new remedy at first, and reluctantly adopted by the French physicians, on Dr. Conolly's representation.

Evanson and Maunsell, who advise mercurials, do so with a caution against worse effects by dangerous salivation.

So also of its employment at a later stage. I cannot avoid thinking it especially ill-judged to mercuralize a patient—if wise at any stage—at the somewhat advanced period at which the croupal symptoms usually appear in diphtheria, as the peculiar adynamic condition also is approaching, if not already begun, which must be hastened and aggravated by a mercurial course.*

The Sulphate of Quinia is a valuable medicine, and is used by some in preference to Chlorate of Potash. I have not been in the habit of combining them. Mr. Jennings, of Malmesbury, England, prescribes Quin. Sulph., Potass. Chlor. et Acid. Hydrochl., in combination, every four hours. If chlorate of potash has as good an effect, it has one great advantage over quinine—that a child will take it more willingly. Mr. Bottomley, of Croydon, Kent, also recommends quinine, which he added to the chlorine mixture, after the third day, in an epidemic which he treated with great success.

Turpentine is another stimulant that has met with favor. Dr. E. Perry, of Marden, Kent, advises for a child between two and six years old, two minims of Oleum Terebinthinæ every second hour, and five grains of Ammoniæ Carbonas every second hour—the child taking the turpentine one hour

* The correct views on the nature and treatment of typhoid fever, which are extending more widely every year, are gradually eradicating the mischievous practice of mercurialization, which has been a part of the established treatment of fever, but even among those physicians who read and think, and entertain better ideas, we find some who do not dare to withhold the calomel, when the tongue is getting dry and black. As in fever, so in diphtheria, it is not mercury, but turpentine, quinine, and wine, that the dry, black tongue, (with its typhoidal concomitants,) wants.

and the ammonia the next—especially in cases where the larynx has become involved. He says, "I was induced to try the turpentine from having noticed its effects when given as advised by Dr. Carmichael in cases of iritis in broken-down constitutions, where mercury could not be used; and where there is so great a tendency to the effusion of lymph in the chambers of the eye. We all know, too, how effectual it is in other diseases, acting like mercury in many respects—but stimulating instead of debilitating—and hence its appropriateness in diphtheria, where mercury, I believe, hastens to fatal result." He makes no caustic or other application to the fauces—but sustains the patient with wine, porter, and beef tea.

Besides quinine, chloric, or turpentine, diphtheria requires alcoholic stimulants. The flagging powers of life need frequently repeated excitants, to sustain them against the steadily depressing influence of the disease. As soon as the febrile action has subsided—which is usually present during the first day, but hardly ever continues beyond the third—some form of alcoholic stimulant may be safely and usefully administered. It is better to begin it then, than to wait for symptoms of collapse. The absence of heat, the weakness of the pulse, and the tolerance of the first carefully administered drams, will be sufficient indications of the necessity. But we might without hesitation say that the presence of the disease is alone a sufficint indication. The only guide to the amount is its effect in each case; there is usually very great tolerance of wine, brandy, or whatever form can be most easily administered. There is only one writer—Mr. Jennings, already quoted—who has any doubt of the usefulness and necessity of alcoholic stimulation in all cases in which there is any depression. This gentleman, who claims great success in his cases, which have been treated very actively, advises "quinine in large doses, and the avoidance or guarded use of alcoholic stimulants."

There is no specific for diphtheria, and the advantages of the various agents which have been described are not claimed as such. Several of them have been almost universally employed, and with favorable effect in proportion to the curability of the cases. But it is not to be denied that many of the cases might have done equally well on other remedies than those which were used, so that the general plan of treatment was preserved. It is possible that quinine may in many cases take the place of alcohol; but certainly the most malignant cases require a very free use of wine or rum. In different places we find a sufficient variation in the disease to make it probable that the indications are not always the same; it is doubtless attended with less depression in some places than others. Therefore, any individual opinion like this of Mr. Jennings, must be received with allowance. Most writers express very strongly the same opinions that I have advanced. Thus Dr. Perry, already quoted, says, "besides the medicinal treatment, the child takes port-wine, porter, and beef-tea, or wine with the yolk of an egg, *ad libitum*." Mr. Cammack, of Boston, England, "A little mutton, every day; boiled milk, rich gruels, and beef-tea, with hot port-wine and water, for all above ten years; and warm milk and water for minors. All things should be taken warm." Mr. Bottomley, "The diet consists of concentrated jellies, strong beef-tea, wine, etc." Mr. C. S. Smith, "the diet to consist of strong beef-tea, port-wine, and, in short, all the nourishment the patient can take." Mr. Thos. H. Smith says, in the British Med. Journal, "I need not dwell upon the necessity of wine, beef-tea, etc. In the severe cases, they are most urgently required, and must be liberally supplied. In the more trifling cases, if well-marked, convalescence will be delayed, and danger of relapse continue, if these, or their equivalents, are not employed." Mr. McDonald, of Bristol, says, "After a clearance of the bowels with calomel and rhubarb, I order strong beef-tea, wine, and above all, Boss's pale ale; the patients express themselves much relieved in the throat as it is swallowed, and feel greatly exhilarated after taking it. One gentleman drank twelve pint bottles of it in the course of a night." Dr. R. W. Crighton, of Scotland, says, "I had not treated many cases before I found the necessity of resorting (in most cases from the very commencement) to stimulants, and the most strongly nutritive diet, as brandy and beef-tea; and these, in general, could not be withdrawn until an advanced period of the convalescence." (*Edinboro' Med. Journal*, *Feb.* 1860.)

When solid food can no longer be taken, beef juice, prepared according to Liebig's formula, furnishes the most concentrated nutriment that is available—to which may be added whey or strong porridge for variety. Difficult deglutition very frequently makes it impossible to take the necessary amount of nourishment. In this case we should, without delay, resort to its administration by the rectum. It is very common to make use of nutritive enemata only *in extremis;* but they are given with so great ease and no inconvenience, and supply so well the wants of the system, that there is no need of waiting a single hour after the sufficient amount cannot be taken by the mouth. I have found that two or three ounces of beef juice can be retained and absorbed by a child every five or six hours; and a smaller quantity will be enough for a very young child. One ounce every four hours—six ounces a day—(equivalent to the juice of two pounds of beef,) will usually be sufficient for a child of three years of age, in addition to the alcohol it takes. And, if necessary, alcohol diluted can be given by the rectum as well as the beef juice. It is well to add a few drops of laudanum to the enerma—but after two or three enemata, they will generally be retained without it.

Blisters are objectionable, on account of their liability to become affected with diphtheric exudation. And, even without this danger, there is no evidence of their giving any relief.

Dr. Bostwick, of Red Rock, N. Y., excises a portion of the uvula, which he finds very long and œdematous, and has found the operation check the local affection very materially.

Excision of the surface of the tonsils has been advised, but I am unable to say with what success it has been practised; if such treatment were capable of removing the disease, it would furnish the best evidence that it originates locally, as some suppose.

Tracheotomy has been performed in cases where croupal symptoms arose, and has been successful in several, but in an extremely small number of instances. The constitutional disease which underlies the laryngeal affection, places it of course in a very different category from croup, and renders a fatal termination almost inevitable. Dr. Seymour, of Troy, says in a private letter, "the few cases I have seen (of the croupal form) impressed me with the idea of far less physical obstruction than exists in a case of inflammatory croup; for though the voice might be very much smothered and husky, and æration interfered with, there was not that sibilant or shrill sound to the voice, and it will be at times quite loud and distinct, though the forced respirations in crying are at the same time husky. The effect of the difference, in my judgment, was to this opinion—that tracheotomy was not indicated nor could be of the same service as in inflammatory croup The parts seemed more poisoned than plugged." The false membrane is not found after death in these cases so thick as in membranous croup; forming in the larynx usually at a late period, therefore of fewer laminæ and less consistence than on the fauces; and consequently the calibre of the larynx is less diminished than in croup. Yet in the enfeebled condition of the patient, we can easily believe that no obstruction to respiration can be safely borne; hence it does not surprise us that cases in which the croupal symptoms are present, are almost invariably fatal.

Besides the constitutional state, it is another objection to tracheotomy that the false membrane frequently extends through the trachea and a considerable portion of the bronchi, rendering the operation fruitless; a condition that exists in membranous croup indeed, but less often, I believe. Dr. Bontecou, of Troy, operated on a child, who lived five days afterwards. At the *post-mortem* examination "the exudation was found extending down into the bronchial ramifications of one lung, which was hepatised."

To express in a word the present state of the question of tracheotomy, Trousseau now makes it one of several conditions of operating in membranous laryngitis, that the disease shall not be diphtheric. (*Champonniere's Journal*, 1859.) In diphtheria, the operation is almost invariably followed by death; while in membraneous croup, Trousseau states that of late years, since he had adopted the plan of operating before the circulation is seriously embarrassed, half his cases of tracheotomy in private practice have recovered.

The local applications, the tonics and the stimulants, important as they are, are fully equalled in necessity by measures for free ventilation and cleanliness. There can be no doubt that not only is the disease rendered infectious by the neglect of these precautions, but the severity of the cases already under treatment is very much increased. There is in the medical profession generally, about the same amount of belief in the value of free ventilation and cleanliness that will be found in the best informed half of the community—a theoretical, not a practical belief in the standard sanitary doctrines. For I believe it will be admitted that very few, even medical men, look upon these means as anything more than *juvantia:* their directions for the ventilation of sick rooms are seldom given with any approach to the exactness and sense of their importance, which are employed in prescribing medicines or diet. The history of diphtheria makes it clearer than day that no cause is more effective in spreading it and making it virulent, than crowding and foul air. Segregation of the sick, and the most thorough and unintermitting change of the air of the room and house, are absolutely indispensable.

The directions of Nathan Smith for the hygienic treatment of typhoid fever, are applicable to diphtheria. An open chimney with a fire in it, is the first indispensable. The bed must be so arranged that the air from out-of-doors shall blow over and around it all the time.* Nurses require to be repeatedly assured that the patients will not "get cold" in bed, if sufficiently clothed, and the attendants must protect themselves. And the more bare the room is of furniture, especially of drapery or any kind of stuffs, the fewer media of infection there will be. The bed-clothes must be changed daily, and thoroughly aired, morning and night, while the patient is removed to another bed.† At the same time the danger of fatal syncope should not be forgotten at the advanced stage of diphtheria, and the erect position should be studiously avoided. And, lastly, thorough daily ablution of the whole body is imperatively necessary.

Diphtheria cannot be considered a disease whose treatment is uncertain, or still merely matter of experiment. We are justified, by all experience, in holding clear views of the manner of meeting its indications. It is no condemnation of the method usually pursued, that it is fatal in so large a number of cases. As in typhus, typhoid, scarlet, and congestive fevers, a varying proportion of cases will occur in every epidemic of so virulent and malignant a nature that medical treatment is unavailing. The best methods, applied even at the outset, have no effect in saving life or mitigating the severity of the symptoms. Then, casting out such cases, there are many others which are fatal from being neglected until the disease

* Florence Nightingale's little book on Nursing, to which her great name has given the currency it deserves, as perhaps the most thorough and earnest book of the kind that ever was written, is full of good doctrine, and delivered with a directness of personal appeal, that scientific conventionality often forbids medical men to use. It is a book not merely for the people, but a true guide to professional men, sound in hygienic teaching, and especially applicable to such diseases as the one now under consideration. If some of her views on the spontaneous origin of zymotic diseases are not sound, they are not dangerous, but quite the reverse. No man need be ashamed to strengthen his hands by her authority; and no one who has earnestly striven all his life to impress upon those who come under his influence, the superlative value of hygienic agencies, in the prevention and treatment of diseases, and especially in the staying of pestilences, can fail to welcome her powerful aid to his cause.

† The most successful treatment on record of any zymotic disease of a serious character, is purely hygienic. It is the case of eighty-two people sick with typhus of a grave character—twelve of them already insensible—who were landed in 1837, on the New Jersey shore, from a wreck, and laid in canvas shanties, exposed to the weather, and drenched with rain, but not otherwise treated, except with cordials—*and all recovered.* No hospital records can show such a result. (*Sickness and Mortality on Emigrant Ships. Cong. Report.*) It is not to be supposed that a similar course would deprive an epidemic of diphtheria of all its malignancy—but to what extent it would diminish it, we can never know, till a much more thorough hygiene than is common, has been fairly tried.

is far advanced that the opportunity for medical applications has passed. Diphtheria is clearly not a self-limited disease; and in order to make treatment effectual, it must be begun at an early period. And, as it is evident that from ignorance or neglect on the part of patients and their friends, many are not seen by a physician till the disease has continued for several days, it is proper, in judging of the value of the medical treatment, that all such cases should be left out of the consideration. Such a condition applies equally, of course, to all varieties of medical treatment. But in the case of the disease we are now examining, it has already been observed that in the essentials there is little variety in the methods which have been adopted for its treatment; and therefore the question is not at present one of comparison between this treatment and any other. It is only a question whether, when one patient in ten, eight or five, dies under a certain mode of treatment, that treatment can be judicious. I repeat, that, to judge it fairly, we must diminish the number of fatal cases, by a few exceedingly malignant and many that doubtless remained without treatment until the stage had passed in which any impression could be made on the disease.—*Berkshire Medical Journal.*

